# Supplementary material for: Target product profiles for new diagnostics to inform strongyloidiasis control programs
Source: PLoS Negl Trop Dis. 2025 Jul 7;19(7):e0012774. doi: 10.1371/journal.pntd.0012774 (PMC12251137; doi:10.1371/journal.pntd.0012774)
Supplement: S1 Table — (DOCX) [file pntd.0012774.s001.docx]

**S1 Table. Values of the required parameters to assess the risk of incorrect decision-making when currently available test platforms are deployed.**

| **Parameters** | **Description** | **Baermann method** | **Ab-based assays** |
| --- | --- | --- | --- |
| $n_{schools}$ | Number of schools | 5 - 10 | |
| $n_{children}$ | Number of children per school | 10 - 100 | |
| $se$ | Diagnostic sensitivity of the test | 0.50 | 0.79 |
| $sp$ | Diagnostic specificity of the test | 0.98 | 0.93 |
| $T$ | Program prevalence threshold | 0.10 | |
| $LL$ | Lower limit of the grey zone | 0.075 | |
| $UL$ | Upper limit of the grey zone | 0.125 | |
| $ICC$ | Intra-cluster correlation | 0.0014 | |
