# Supplementary material for: Target product profiles for new diagnostics to inform strongyloidiasis control programs
Source: PLoS Negl Trop Dis. 2025 Jul 7;19(7):e0012774. doi: 10.1371/journal.pntd.0012774 (PMC12251137; doi:10.1371/journal.pntd.0012774)
Supplement: S1 Info — (DOCX) [file pntd.0012774.s002.docx]

**Target product profiles for new diagnostics to inform strongyloidiasis control programs**

**S1 Info. Quantification of spatial heterogeneity in strongyloidiasis**

Adama Kazienga^1,2*^, Luc E. Coffeng^2^, Sara Roose^1^, Sake de Vlas^2^, Dora Buonfrate^3^ Salvatore Scarso^3^, Francesca Tamarozzi^3^, Bruno Levecke^1^

^1^Department of Translational Physiology, Infectiology and Public Health, Ghent University, Merelbeke, Belgium

^2^Department of Public Health, Erasmus MC, University Medical Center Rotterdam, Rotterdam, The Netherlands

^3^ Department of Infectious, Tropical Diseases and Microbiology, IRCCS Sacro Cuore Don Calabria Hospital, Negrar di Valpolicella, Verona, Italy

*Corresponding author: [kazienga_adama@yahoo.fr](mailto:kazienga_adama@yahoo.fr)

This supplement describes how we estimated the spatial heterogeneity strongyloidiasis across schools (intra-cluster correlation). For this, we re-analysed the data of a previously conducted survey in Ethiopia. This study was designed to determine the prevalence of strongyloidiasis in Ethiopian school-aged children (SAC). The dataset included test results for 6,846 SAC from 64 schools and 33 woredas (administrative units). In the following paragraphs will provide more details on (i) the ethical procedures, (ii) the applied assay to assess strongyloidiasis infections and (iii) the methodology deployed to estimate the spatial heterogeneity.

**Assays to determine the presence strongyloidiasis**

To determine the presence of strongyloidiasis in the SAC, the InBios Strongy Detect^TM^ IgG ELISA was deployed on serum samples as described by Tamarrozi et al. [2], resulting in an overall prevalence in strongyloidiasis of 2.3%.

**Estimate the spatial heterogeneity in strongyloidiasis.**

To estimate the spatial heterogeneity in strongyloidiasis, we fitted a generalized linear mixed model using the abovementioned dataset, accounting for school and woreda effects. The specification of the model was as follows:

$Y_{ijk} \sim Bernoulli (p_{ijk})$ Eq S1.1

$logit \left( p_{ijk} \right)= \beta_{0}+d_{k}+s_{jk}$ Eq S1.2

$$d_{k} \sim N (0, \sigma_{k}^{2})$$

$$s_{jk} \sim N (0,\sigma_{jk}^{2})$$

Here, $Y_{ijk}$ represents the binary outcome for student $i$ in school $j$ within woreda $k$, $p_{ijk}$ is the probability that student $i$ is infected with strongyloidiasis, $d_{k}$ is the random effect for woreda $k$, and $s_{jk}$ is the random effect for school $j$ within woreda $k$, $\sigma_{k}^{2}$ represents the woreda-level variance and $\sigma_{jk}^{2}$ is the school-level variance.

We further estimated the intra-cluster correlation using the following formula:

$$ICC= \frac{\sigma_{jk}^{2}}{\sigma_{k}^{2}+ \sigma_{jk}^{2}+ \sigma_{e}^{2}}$$

Here, $\sigma_{e}^{2}$ represents the residual variance for logistic regression and was set at $\pi^{2}/3$ [3], based on the logistic distribution. Using this model, the estimated variance for the woreda level was 1.0264, the estimated variance for the school level was 0.0063, resulting in an intra-cluster correlation of 0.0014.

**References**

1. Roose S, Leta GT, Vlaminck J, Getachew B, Mekete K, Peelaers I, et al. Comparison of coproprevalence and seroprevalence to guide decision-making in national soil-transmitted helminthiasis control programs: Ethiopia as a case study. PLoS Negl Trop Dis. 2022;16: e0010824.

2. Tamarozzi F, Longoni SS, Mazzi C, Pettene S, Montresor A, Mahanty S, et al. Diagnostic accuracy of a novel enzyme-linked immunoassay for the detection of IgG and IgG4 against Strongyloides stercoralis based on the recombinant antigens NIE/SsIR. Parasit Vectors. 2021;14: 1–11.

3. Adam NS, Twabi HS, Manda SOM. A simulation study for evaluating the performance of clustering measures in multilevel logistic regression. BMC Med Res Methodol. 2021;21: 1–14.
