# Supplementary material for: Target product profiles for new diagnostics to inform strongyloidiasis control programs
Source: PLoS Negl Trop Dis. 2025 Jul 7;19(7):e0012774. doi: 10.1371/journal.pntd.0012774 (PMC12251137; doi:10.1371/journal.pntd.0012774)
Supplement: S2 Info — (DOCX) [file pntd.0012774.s003.docx]

**Target product profiles for new diagnostics to inform strongyloidiasis control programs**

**S2 Info. Estimation of the total survey cost**

Adama Kazienga^1,2*^, Luc E. Coffeng^2^, Sara Roose^1^, Sake de Vlas^2^, Dora Buonfrate^3^ Salvatore Scarso^3^, Francesca Tamarozzi^3^, Bruno Levecke^1^

^1^Department of Translational Physiology, Infectiology and Public Health, Ghent University, Merelbeke, Belgium

^2^Department of Public Health, Erasmus MC, University Medical Center Rotterdam, Rotterdam, The Netherlands

^3^ Department of Infectious, Tropical Diseases and Microbiology, IRCCS Sacro Cuore Don Calabria Hospital, Negrar di Valpolicella, Verona, Italy

*Corresponding author: [kazienga_adama@yahoo.fr](mailto:kazienga_adama@yahoo.fr)

# Introduction

This document describes the cost-analysis to determine the total survey costs surveys when deploying the Baermann method, Ab-ELISA (based on the analysis of either plasma (Ab-ELISA/plasma) or dried blood samples (Ab-ELISA/DBS)). Generally, the total survey cost for school-based survey comprises of five cost items: (i) the operational cost to inform all schools ($Cost_{inform}$), (ii) the cost to collect and prepare all individual samples for further analysis ($Cost_{collect}$), (iv) the cost to transport all samples to a central laboratory ($Cost_{transport}$), and (v) the cost to analyze all samples ($Cost_{analysis}$), resulting in the following formula:

$Cost_{tot}=Cost_{inform} + Cost_{collect} + Cost_{transport}+ Cost_{analysis}$ **Eq (1)**

In the following sections we will describe these four cost attributes in more details.

#### **Cost to inform the schools**

This cost concerns the time required to inform all the schools of the purpose of the study and is based on the number of schools ($n_{schools}$), the number of laboratory technicians ($n_{tech}$), their daily salary ($Cost_{tech}$) and the cost of daily car rental and driver wage ($Cost_{drivers}$). This cost was then defined as follows:

$Cost_{inform}=n_{schools}\times(n_{tech} \times Cost_{tech} +Cost_{drivers})$ **Eq (2)**

# Cost to collect and prepare samples

The cost to collect and process all samples, is the sum of the cost for consumables to collect and prepare all samples for further analysis ($Cost_{collect\_consumables}$) and to actually collect and prepare all samples ($Cost_{collect\_operation}$). The formula for $Cost_{collect\_consumables}$ can be written as

$Cost_{collect\_consumables}= n_{schools}\times n_{children} \times Cost_{collect}$ **Eq (3)**

In this formula, $n_{schools}$represents the number of schools, $n_{children}$, the number of children per school, $Cost_{collect}$, the cost of consumables to collect a single sample per child.

$Cost_{collect\_operation}$ includes the number of days to complete a survey ($n_{days}$), the number of laboratory technicians ($n_{tech}$), and their daily per diem ($Cost_{tech}$), the cost of car rental, gasoline and drivers wage ($Cost_{drivers}$) to transport the team. Note that an additional cost of local helper is needed when deploying the Baermann method. We assumed that the local helper worked all days except the last day of the survey. For this, we added this cost using an indicator ‘Baermann’ (1 if a Baermann-based survey and 0 otherwise). We defined the operational cost for collection and processing samples as follows:

${Cost_{collect\_operation}=n}_{days}\times\left( n_{tech} \times Cost_{tech}+Cost_{drivers} \right) +Baermann \times Cost_{helper}\times\left( n_{days}-1 \right)$ **Eq (4)**

The number of days needed to complete a survey ($n_{days}$) can be written as below

$n_{days}= \frac{n_{schools} \times n_{children}}{n_{tech} \times n_{hours} \times n_{throughput\_collect}}$ **Eq (5)**

In this formula, $n_{tech}$represents the number of laboratory technicians, $n_{hours}$, the number of daily working hours, and $n_{troughput\_collect}$, the number of samples per hour that can be collected by one person.

# Cost to transport the samples to a central laboratory

For the Ab-ELISA-based surveys, samples are transported to a central laboratory. This transport cost ($Cost_{transport}$) will depend on the total number of schools included in the survey ($n_{schools}$), the number of schools that triggers transport of samples to the central laboratory ($max_{schools}$), the time (in days) it takes to transport the samples to the central laboratory by one laboratory technician ($n_{days\_trans}$), the salary for this laboratory technician ($Cost_{tech}$) and the cost for car rental, gasoline and drivers wage ($Cost_{drivers}$).

$Cost_{transport}= \frac{n_{schools}}{max_{schools}}\times n_{days\_trans}\times\left( Cost_{tech}+Cost_{drivers} \right)$ **Eq (6)**

# Cost to analyze samples

The cost to analyze all samples, is the sum of the cost for consumables to analyze all samples ($Cost_{analysis\_consumables}$) and to actually analyze all samples ($Cost_{analysis\_operation}$). The formula for $Cost_{analysis\_consumables}$ was defined as follows:

$Cost_{analysis\_consumables}= n_{schools}\times n_{children}\times\left( ELISA \times Cost_{prep}+Cost_{test} \right)$ **Eq (7)**

Here, $n_{schools}$represents the number of schools, $n_{children}$, the number of children per school, $ELISA$ is an indicator for the implementation of an Ab-ELISA at the central laboratory (1 when Ab-ELISA/plasma or Ab-ELISA/DBS are deployed and 0 when the Baermann method or LFA are used), $Cost_{prep}$, the cost to prepare samples for the Ab-ELISAs, $Cost_{test}$the cost per test to analyze one sample.

Finally, we estimated the operating cost to analyze samples ($Cost_{operational\_analysis}$). This cost included the number of days needed to complete the analysis ($n_{days\_analysis}$), the daily per diem of an Ab-ELISA expert ($Cost_{\exp\_tech}$), the number of laboratory technicians ($n_{tech}$) and their daily salary (${Cost}_{tech}$). It also includes two indicators: $ELISA$ (1 when Ab-ELISA/plasma or Ab-ELISA/DBS are deployed and 0 when the LFA is used) and LFA (1 when LFA is deployed and 0 otherwise). We defined the operational cost to analyze samples as follows:

$Cost_{analysis\_operational}= n_{days\_analysis}\times\left( ELISA \times n_{\exp\_tech} \times Cost_{exp\_tech}+LFA\times n_{tech} \times{Cost}_{tech} \right)$ **Eq (8)**

$n_{days\_analysis}= \frac{n_{schools} \times n_{children}}{ELISA \times\left( n_{\exp\_tech} \times n_{hours} \times n_{trougput\_ana} \right) +LFA \times\left( n_{tech} \times n_{hours} \times n_{troug\_ana} \right)}$

**Eq (9)**

Here, $n_{schools}$represents the number of schools, $n_{children}$, the number of children per school, $n_{hours}$ the number of daily working hours, $n_{troughput\_ana}$ the number of samples that can be analyzed by one person in 1 hour.

**Table A** shows the monetary values for each required cost item. These values were based on study designed to compare different diagnostic assays (e.g., Baermann method, qPCR, Ab-ELISA and LFA) in an Ecuadorian context [1].

**Table A. Overview of the cost parameters for the Baermann method Ab-based assays in an Ecuadorian setting.**

| **Variables** | **Description** | **Baermann method** |  | **Ab-based assays** | | |
| --- | --- | --- | --- | --- | --- | --- |
|  |  |  |  | Ab-ELISA/ Plasma | Ab-ELISA/  DBS | LFA |
| $n_{tech}$ | Number of laboratory technicians | 4 |  | 2 | 2 | 2 |
| $n_{hours}$ | Number of daily working hours | 4 |  | 8 | 8 | 8 |
| $n_{troughput\_collect}$ | Number of samples collected per hour | 6 |  | 4 | 16 | 8 |
| $n_{\exp\_tech}$ | Number of ELISA experts | 0 |  | 1 | 1 | 0 |
| $n_{troughput\_ana}$ | Number of samples that can be analyzed by one person in 1 hour | 6 |  | 11 | 11 | 11 |
| $Cost_{tech}$ | Per diem for laboratory technicians for one day (in EUR) | 63 |  | 63 | 63 | 63 |
| $Cost_{drivers}$ | Cost for car rental and driver for one day (in EUR) | 110 |  | 110 | 110 | 110 |
| $Cost_{helper}$ | Per diem for local helper for one day | 12.5 |  | 0 | 0 | 0 |
| ${Cost}_{collect}$ | Cost to collect one sample (in EUR) | 0.65 |  | 2.53 | 3.05 | 1.19 |
| $Cost_{\exp\_tech}$ | Per diem for an ELISA expert for one day (in EUR) | 0 |  | 63 | 63 | 0 |
| $Cost_{prep}$ | Cost to prepare one sample for Ab-ELISA | 0 |  | 9.27 | 11.49 | 0 |
| $Cost_{test}$ | Cost to test one sample | 1.77 |  | 10.79 | 10.79 | 2.09 |
| $n_{days\_trans}$ | Number of days to carry samples to a central lab | 0 |  | 2 | 2 | 0 |
| $max_{schools}$ | The number of schools that triggers transport of samples to the central laboratory | 0 |  | 10 | 10 | 0 |

# Reference

1. Tamarozzi F, Guevara ÁG, Anselmi M, Vicuña Y, Prandi R, Marquez M, et al. Accuracy, acceptability, and feasibility of diagnostic tests for the screening of Strongyloides stercoralis in the field (ESTRELLA): a cross-sectional study in Ecuador. Lancet Glob Heal. 2023;11: e740–e748.
