# Supplementary figures and images for: Target product profiles for new diagnostics to inform strongyloidiasis control programs
Source: PLoS Negl Trop Dis. 2025 Jul 7;19(7):e0012774. doi: 10.1371/journal.pntd.0012774 (PMC12251137; doi:10.1371/journal.pntd.0012774)

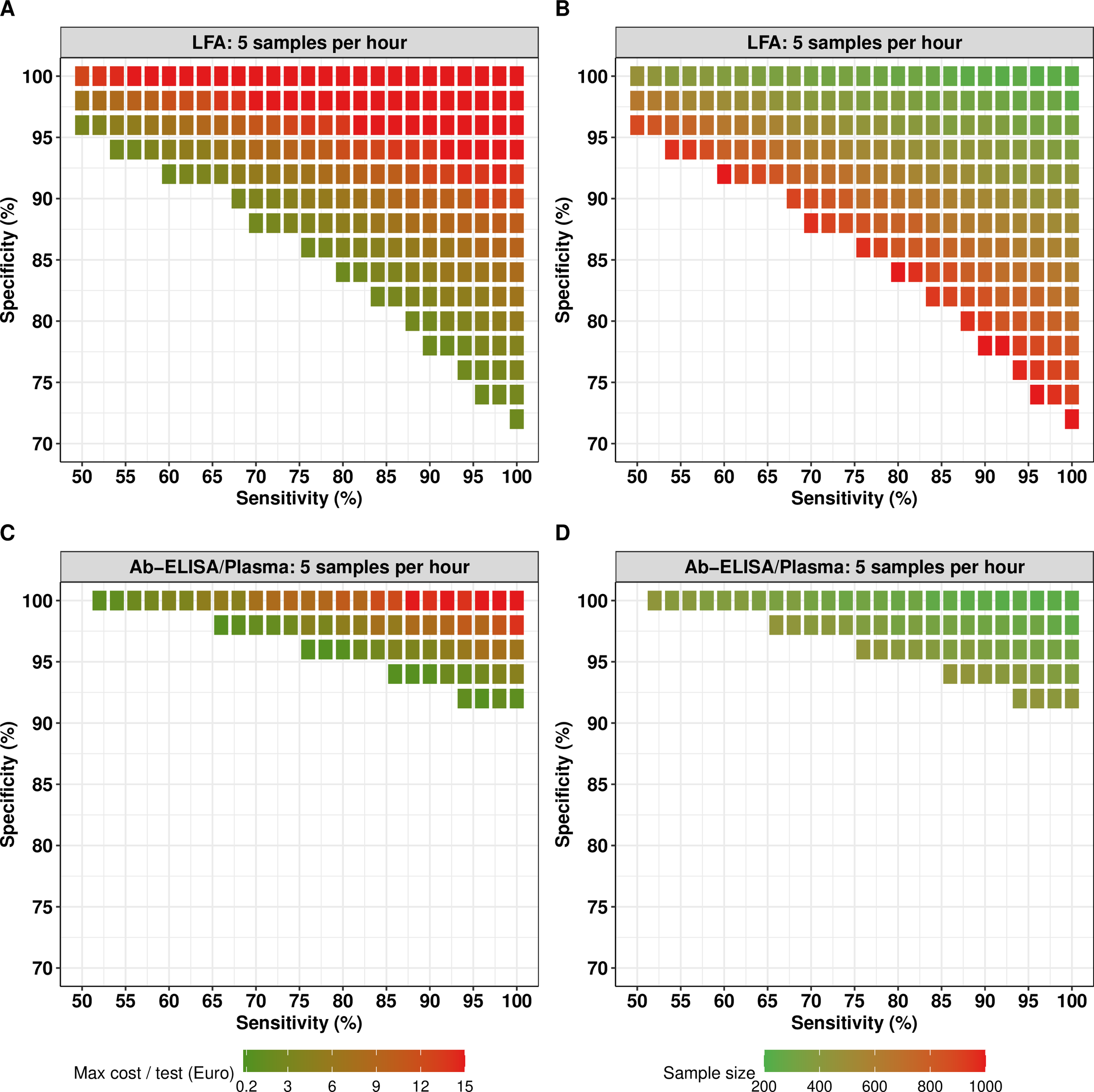

Supplement: S1 Fig — This figure represents all possible combinations of sensitivity and specificity and the maximum cost per test (in EUR) (Panel A: LFA and Panel C: Ab-ELISA/Plasma) and the required sample size (Panel B: LFA and Panel D: Ab-ELISA/Plasma) that allowed for adequate (Eundertreatment=5% and Eovertreatment=25%) decision-making. Note that the maximum cost per test is the true maximum cost per test that could work in at least one logistically feasible survey design that is not more expensive than the benchmark cost based on the Baermann method. Also, the required sample size corresponds to the minimum required number of schools and children per school that need to be sampled for each combination of sensitivity and specificity. Absence of tiles indicates that the combination of sensitivity and specificity was inadequate for decision-making, given the logistic and budget constraints. (TIF) [file pntd.0012774.s005.tif]
